# Amyloid Cascade Hypothesis for the Treatment of Alzheimer’s Disease: Progress and Challenges

**DOI:** 10.14336/AD.2022.0412

**Published:** 2022-12-01

**Authors:** Tong Wu, Ding Lin, Yaqian Cheng, Senze Jiang, Muhammad Waheed Riaz, Nina Fu, Chenhao Mou, Menglu Ye, Ying Zheng

**Affiliations:** ^1^State Key Laboratory of Subtropical Silviculture, Zhejiang A&F University, Hangzhou 311300, China; ^2^Zhejiang Provincial Key Laboratory of Resources Protection and Innovation of Traditional Chinese Medicine, Zhejiang A&F University, Hangzhou 311300, China

**Keywords:** Alzheimer’s disease;, amyloid cascade hypothesis, drug development strategy, clinical trials

## Abstract

The amyloid cascade hypothesis has always been a research focus in the therapeutic field of Alzheimer’s disease (AD) since it was put forward. Numerous researchers attempted to find drugs for AD treatment based on this hypothesis. To promote the research of anti-AD drugs development, the current hypothesis and pathogenesis were reviewed with expounding of β-amyloid generation from its precursor protein and related transformations. Meanwhile, the present drug development strategies aimed at each stage in this hypothesis were also summarized. Several strategies especially immunotherapy showed the optimistic results in clinical trials, but only a small percentage of them eventually succeeded. In this review, we also tried to point out some common problems of drug development in preclinical and clinical studies which might be settled through multidisciplinary cooperation as well as the understanding that reinforces the amyloid cascade hypothesis.

## 1.Background

Alzheimer’s disease (AD), whose clinical feature is cognition impairment, was first discovered in the brain of a patient who died due to the progressive decline of brain function by doctor Alzheimer in 1906 [1]. Today, AD is an increasing global health challenge, with 40-50 million people suffering from it. The patient will develop into memory deficiency, language disorders, behavioral and psychological symptoms of dementia (BPSD), and even death. It is estimated that the number of people with AD will reach around 100 million by 2050 around the world [2]. There is no effective treatment for AD so far and drug development for AD is complex and has a high failure rate [3].

The causes of AD are not well defined at present which hinders the research of anti-AD agents. Cholinergic nerve injury is one of the leading theories on the causes of AD pathogenesis. This theory holds that the lack of acetylcholine (Ach), a neurotransmitter, will affect the functions of the hippocampus and cortex, where the brain processes information [4, 5]. At present, most marketed drugs for the treatment of AD are designed based on this theory, such as acetylcholinesterase inhibitors including Donepezil, Galanthamine, and Huperzine A, which improves the cognitive function of patients by slowing the breakdown of acetylcholine in the synaptic cleft and consequently increasing acetylcholine content [6]. Few anti-AD drugs target other neurotransmitters, such as memantine which can non-competitively antagonize N-methyl-D-aspartate (NMDA) receptors [7]. Hyper-phosphorylation tau (HP-tau) is also identified to be one of the causes of Alzheimer's disease. Hyper-phosphorylated microtubule-associated protein tau is the major protein component of neurofibrillary tangles (NFTs) causing neuronal degeneration. The hyperphosphorylation of tau will also disrupt microtubules, which participate in the formation of axons and dendrites of neuronal as an important cytoskeleton system [8]. According to several studies, the imbalance of intestinal flora, the absence of melatonin, and the dysfunction of axonal transport in neurons will also lead to AD [9].

β-amyloid (Aβ) deposition, which was first discovered in cerebrovascular patients in the 1980s, is regarded as the leading cause of AD [10]. After sequencing, Aβ was found to be associated with a variety of genetic mutation related to AD [11]. Aggregation and deposition of Aβ cause the dysfunction of neurons, which leads to the development of AD. At present, Aβ is the most common target in the clinical trial for the treatment of AD [12]. According to the studies, there were 126 agents in clinical trials for AD treatment, and as of 2021, with almost 29% targeting Aβ in phase 3 and 17% in phase 2. However, the clinical trials of drugs targeting Aβ are challenging with a 99% failure rate [13-15]. Some drugs have been able to reduce Aβ at the stage of the clinical trial but most of them did not achieve a significant improvement in cognitive and memory function of AD patients. The drugs in clinical trials which targeted Aβ are listed in Table 1. Although possessing a high risk of failure, Aβ is still regarded as a hopeful target for anti-AD drug development. Aducanumab, a monoclonal antibody of Aβ, was approved by the U.S. Food and Drug Administration (FDA) on June 7, 2021 [16], which encouraged the drug's development aiming at the Aβ pathway. It was also the first novel therapy approved by FDA for AD treatment since 2003. To facilitate the anti-AD drug research, the producing and pathogenic pathway of Aβ and corresponding drug development were systematically reviewed.

## 2.β-amyloid-related pathway and pathogenesis

**Figure 1.**
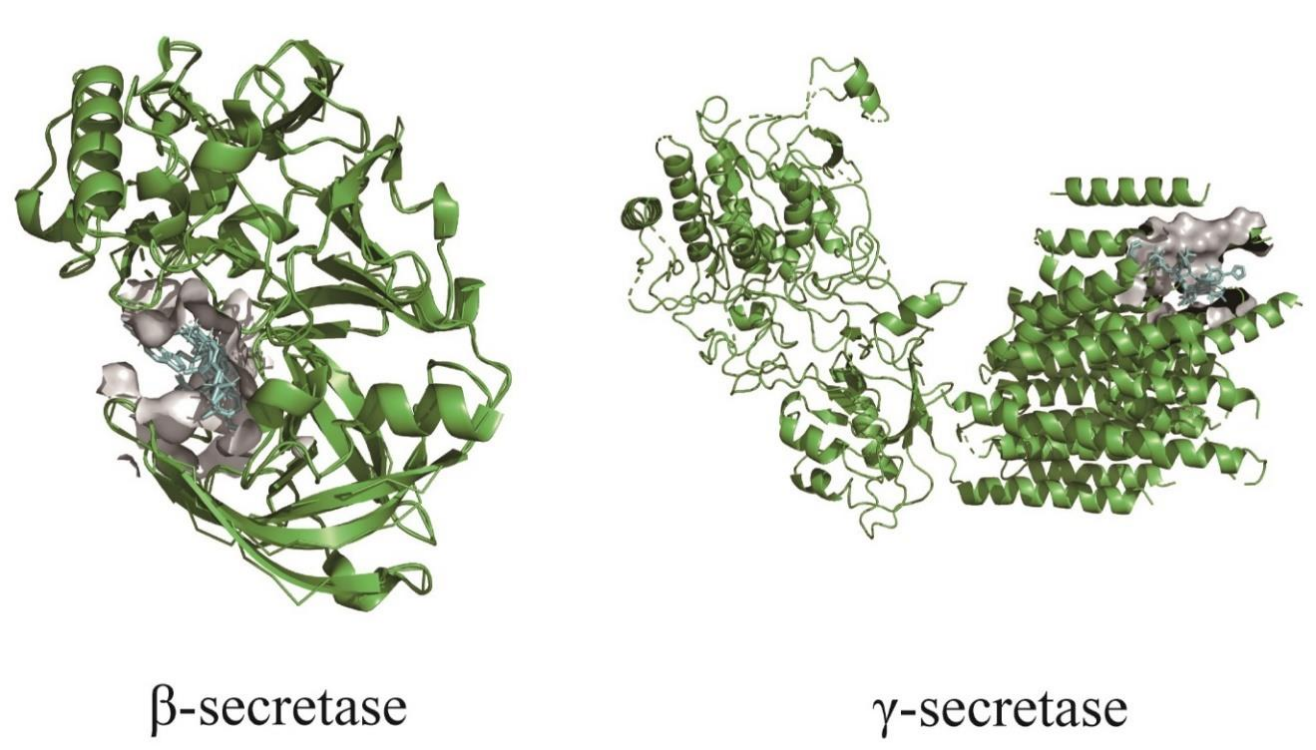
Protein sturctures of β-secretase (RCSB: 2ZHV) and γ-secretase (RCSB: 4UIS), and the interaction with drugs or inhibitors.

The origin of the Aβ pathogenic pathway is mostly considered as the amyloid precursor protein (APP), a kind of transmembrane glycoprotein widely existing on cell membranes of many tissues throughout the whole body. The level of APP expression and activities correlate with the degree of senility [17]. In non-pathological cases, only a small fraction of APPs are degraded into Aβ by β-secretase and γ-secretase in sequence (Fig. 1). However, the mutation of APP encoding genes, which are located on chromosome 21, will generate a new enzymolysis site so that making APP much easier to be cleaved by β-secretase and leading to the increase of Aβ level. The absence of mitochondrial function also affects the expression and process of APP as well as the accumulation of Aβ.

**Table 1:** Drugs targeting at amyloid cascade hypothesis in clinical trials (ClinicalTrials.gov accessed).

Agent	Phase	Mechanism of action
SHR-1707	1	Prevent Aβ plaque and activate microglia to phagocytize various forms of Aβ
BMS-984923	1	Inhibit PrPC-mGluR5 interaction, block pathological Aβ oligomers signal transduction
LY3372993	1	mAb, reduce Aβ
ABvac40	2	Active immunity, remove Aβ
IVIG	2	Antibody, remove amyloid
RO7126209	2	mAb, Anti-Aβ
ALZ-801	2	Prodrug of tramiprostate, inhibits Aβ aggregation
APH-1105	2	Alpha-secretase modulator, reduce Aβ production
Grapeseed Extract	2	Prevent aggregation of Aβ
TEP	2	Activates transport protein ABCC1, remove Aβ
PQ912	2	Glutaminyl cyclase enzyme inhibitor, reduce Aβ production
Gantenerumab	2/3	mAb, Aβ plaques and oligomers
Lecanemab	2/3	mAb, Aβ protofibrils
Donanemab	3	mAb, pyroglutamate form of Aβ
Solanezumab	3	mAb, Aβ monomers
Azeliragon	3	reduce Aβ transport into the brain
Hydralazine hydrochloride	3	Amyloid-binding compounds, accelerate the formation of stable and inert amyloid fibrils

**Figure 2.**
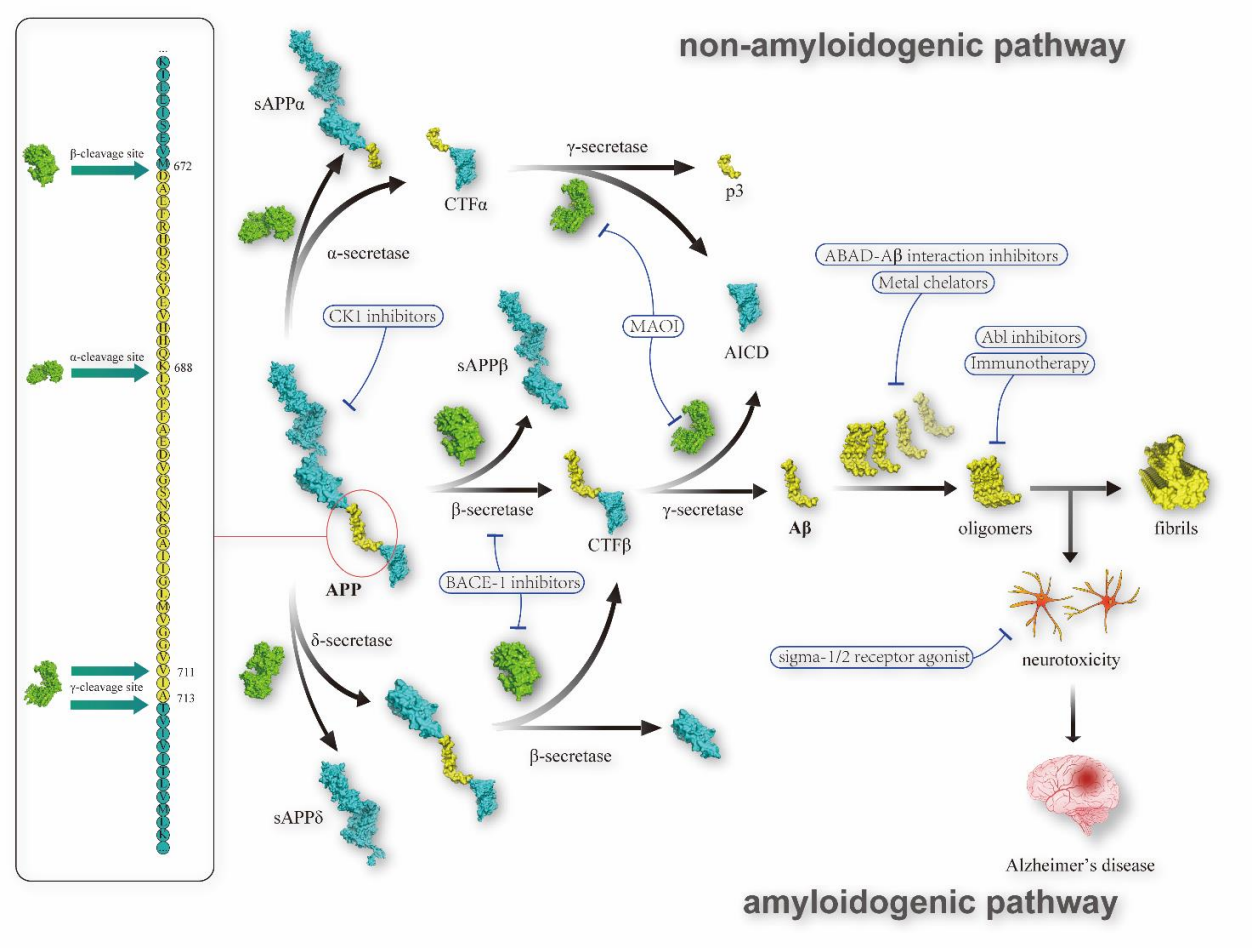
β-amyloid production network and drug development strategies.

On the amyloidogenic pathway (Fig. 2), APP is transformed into Aβ through two-stage enzymolysis. Firstly, the N-terminal of an Aβ sequence is cleaved by β-secretase generating soluble APPβ (sAPPβ) and cytoplasmic tail fragment β (CTFβ). A673T, a coding mutation of the APP gene, could inhibit the β-cleavage of APP and consequently reduce the level of Aβ formation by approximately 40% [18]. CTFβ, which contains 99 amino acids, is operated by γ-secretase subsequently to produce APP intracellular domain (AICD) [19] and several types of Aβ with a sequence length of 36 to 43 amino acids. Amongst them, the fragments consisting of 40 or 42 amino acids are the two most common types of Aβ [20]. The accumulation of Aβ is a progressive process as the levels of Aβ are out of balance in AD patients, and sequentially generate amyloid oligomers, protofilaments, fibrils, and amyloid plaques in the neuropil of the brain [21]. In the past, the ultimate production of amyloid plaque was deemed the major cause of AD symptoms. As more and more research focused on the early stage, new pathogenic factors were found. Compared with fibrils or plaques, the insoluble Aβ oligomers would induce more severe neurological injury. The presence of Aβ fibrils could promote the assembly of free Aβ monomers, thereby rapidly expanding the fibrillated region in Aβ plaques, although it does not exhibit neurotoxicity [22]. Besides insoluble Aβ oligomers, some evidence proved that nonfibrillar soluble Aβ oligomers are likewise neurotoxic, although more research is needed in this aspect.

In recent years, a new branch centering on δ-secretase was discovered participating in the modification of APP. δ-secretase is an asparagine endopeptidase (AEP), which could precut APP at N373 and N585, thereby facilitating the work of β-secretase by decreasing the steric hindrance of APP, and finally increasing the production of Aβ [23-25]. It was also reported that the level of tropomysin-related kinase type B (TrkB) is lower in AD patients. In this respect, δ-secretase is likewise adverse, as it can identify and cleave high-affinity receptor TrkB. TrkB is one of the receptors for neurotrophic factors, which can bind and phosphorylate APP consequently reducing the level of Aβ. The neurotrophic activity of TrkB will be damaged after being cleaved by δ-secretase at N365 and N486/489 residues [24]. More interestingly, Tau can also be cleaved by δ-secretase at N255 and N368 residues into the fragment Tau(1-368), a type of neurotoxic product [26] which could promote the levels of BACE1 activities and generation of Aβ through binding an active BACE1 transcription factor called STAT1 [17]. In addition, it was reported that UNC5C, a netrin-1 receptor, would also be cleaved by δ-secretase [27]. UNC5C could inhibit the production of Aβ and alleviate the AD pathologies after binding with ligand [28, 29]. The hypothesis of δ-secretase has been proved in mice models, as the AD pathologies such as cognitive dysfunctions were weakened by inhibiting the expression of δ-secretase. Research on δ-secretase and corresponding drugs development was emerging recently. Compared with β-secretase and γ-secretase, the short duration and small content could not cover up its potential as an anti-AD target.

In addition, there is a non-amyloidogenic pathway that is parallel and competitive with the amyloidogenic pathway [30-32]. In the non-amyloidogenic pathway, APP is cleaved by α-secretase, a metalloprotease that takes part in a wide range of biological processes [33]. Concretely, α-secretase cleaves APP proximal to the β-secretase site, resulting in the release of soluble APPα (sAPPα) and CTFα. CTFα which is composed of 83 amino acids will be subsequently cleaved by γ-secretase into AICD and a short fragment called p3 [19]. sAPPα is a neuroprotective fragment [34], competitively interacting with β-secretase and preventing the β-secretase mediated APP degradation in the amyloidogenic pathway [35]. In addition, the non-amyloidogenic pathway will directly decrease the level of APP through the enzymolysis of α-secretase. Regulation of the expression and activity of α-secretase might inhibit the amyloidogenic pathway and prevent the progress of AD pathology. However, the regulation mechanism of α-secretase is not entirely clear. A disintegrin and metalloprotease (ADAM) 9, 10, and 17 members of the ADAM protease family, were proved to possess the ability to stimulate the α-secretase activity [33]. And this progress would also be regulated by some stimuli, such as neuropeptide pituitary adenylate cyclase-activating polypeptide (PACAP) and phorbol myristate acetate [30, 36]. Additionally, it was proved that cognitive impairment induced by manganese (Mn) was connected with the expression of α-secretase, APP, and sAPPα, while there was no significant effect on levels of β-secretase [37]. The non-amyloidogenic pathway and α-secretase show great potential but still need further investigation before being the drug target or guiding treatment.

The abnormal Aβ clearance would also cause the accumulation of Aβ and bring trouble. In reality, generation of Aβ was also observed in healthy people. There is a balance between the production and clearance of Aβ, while it’s disordered in the pathological state. ATP-binding cassette transporter A1 (ABCA1) might play an important role in the progress, as the expression level of ABCA1 was proved to affect the Aβ deposition. It was reported that some environmental pollutants such as dichlorodiphenyltrichloroethane (DDT) could inhibit the expression of ABCA1 and slow down the clearance of Aβ by decreasing the level of mRNA and protein of the liver X receptor α (LXRα), an ABCA1 autoregulatory transcription factor [38]. This discovery also reveals the relationship between AD and the environment. Additionally, SHANK-associated RH domain-interacting protein (SHARPIN) is a multifunctional protein with the role of an inflammatory activator, which could regulate the peripheral macrophages, thereby adjusting Aβ degradation and inflammatory mechanisms [39]. The clearance of Aβ depends on many factors. Astrocytes [40], apolipoprotein E (ApoE), and receptors of the low-density lipoprotein receptor (LDL-R) also take part in the process of Aβ clearance. Besides, there was a negative feedback mechanism that the level of Wiskott-Aldrich syndrome protein (WASP) family verprolin homologous protein 1 was down-regulated by AICD. As a result, the production of Aβ was limited [41]. Tau, the downstream pathway of Aβ [42], could also adjust the level of Aβ. It was proved that the deletion of Tau led to the weakening of the Aβ clearance and increasing in plaque deposition [42]. Current research on the Aβ balance mechanism is patchy and fragmentary. However, what is little known is how accurate homeostatic control of the amyloidogenic pathway realize and which process can be intervened for therapy.

The accumulation of Aβ would induce neuroinflammation in the downstream process of the amyloid beta cascade. NLRP3 inflammasome, expressed in the central nervous system (CNS) ubiquitously, was one of the most studied inflammasomes. According to the report, NLRP3 inflammasome participates downstream of the amyloidogenic pathway. Briefly, Aβ could activate the NLRP3 inflammasome. This conclusion was proved and accepted but the progress and the mechanism remain controversial. The activated NLRP3 inflammasome would facilitate the release of active caspase-1, thereby secreting the IL-1β and IL-18 to result in pyroptosis [43]. Even worse, Aβ would induce the pyroptosis and generate the NFTs via NLRP3 inflammasome, and then the neighboring neurons could take up the released tau to activate NLRP3. A negative feedback regulation exists in this progress [44].

## 3.Anti-AD drug development strategies based on the amyloid cascade hypothesis

**Figure 3.**
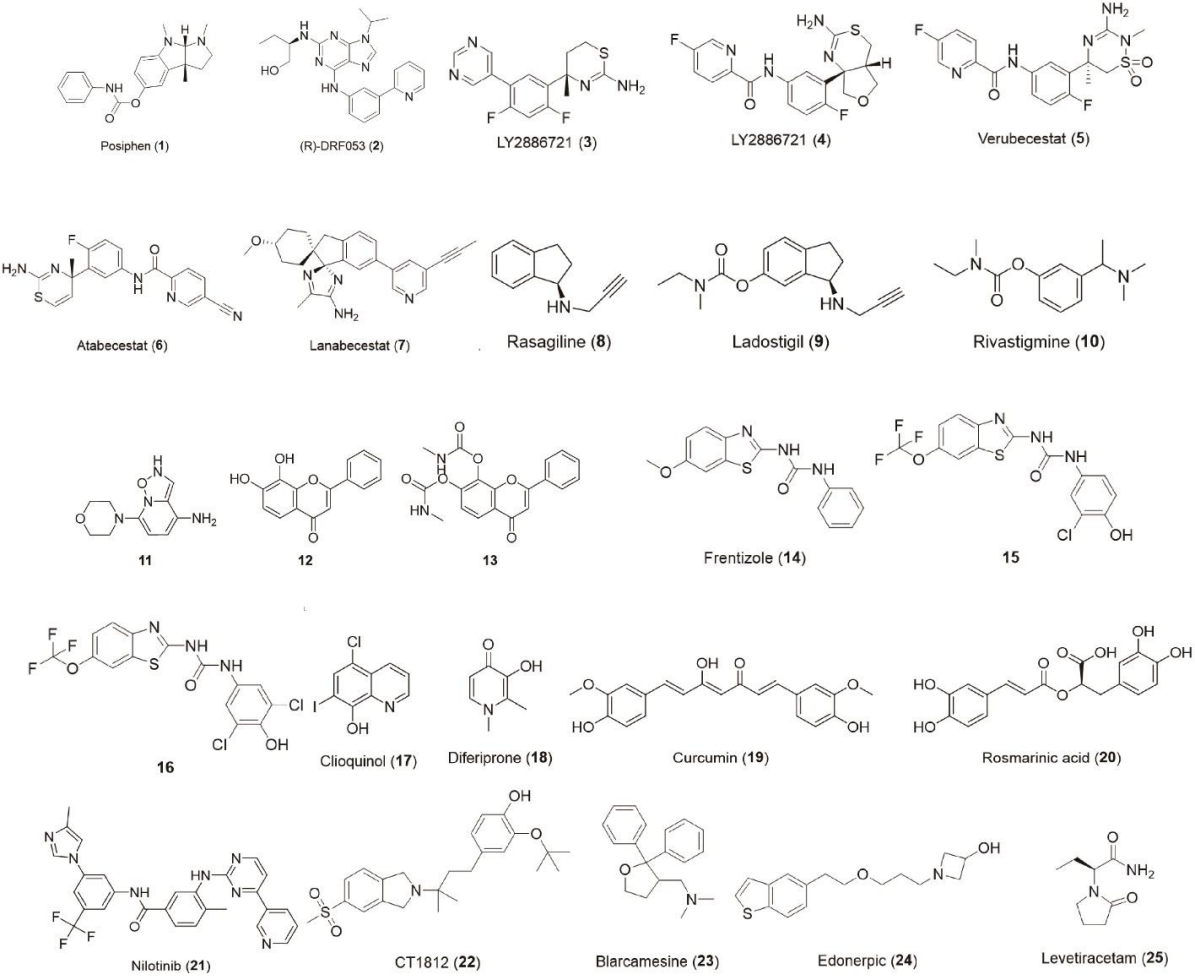
Summarization of drug structures.

The development strategies for anti-AD drugs are classified according to the above pathogenesis. The chemical structures of the drugs involved were displayed in Figure 3.

### 1. Reducing the generation of APP

The APP gene dose hypothesis is a new hypothesis for the treatment of AD, which is associated with the upstream of the amyloidogenic pathway. Based on this hypothesis, the drug development strategy aimed at limiting the level of Aβ by inhibiting the gene expression of APP, which is the precursor of Aβ. Posiphen (1) is supposed to target this hypothesis, and its activity in reducing the translation of APP mRNA has been reported. It proved that Posiphen can reduce the level of APP, Aβ_42_, and related products in mice models. In an ongoing phase I clinical trial, Posiphen can reduce the level of sAPP and cause a decreasing tendency of Aβ_42_ in patients with mild cognitive impairment [45].

Casein kinase 1 (CK1) participates in many cellular process regulations, including the generation of APP. Concretely, CK1, which is derived from astrocytes, is transported to neurons and inhibited the degradation of β-catenin in the form of a complex assembled with neuronal APC and GSK3. β-catenin can be bound to the Hnrnpc gene in the nucleus, which would further promote the translation of the APP gene and the increase of Aβ level [46]. CK1 inhibitors might inhibit the progress and be a new strategy for the treatment of AD [47]. It was reported that the roscovitine derivative (R)-DRF053 (2) possesses potent and dose-dependent CK1 inhibition with an IC_50_ value of 14 nM and low cytotoxicity in the SH-SY5Y cell model [48, 49]. The final activities against the generation of APP of these CK1 inhibitors yet remained for further study. As an upstream hypothesis, the APP gene dose hypothesis provides a new strategy for drug development by cutting off the source of the amyloidogenic pathway.

### 2. Inhibiting the cleavage of APP

In the first step of the amyloidogenic pathway, APP is cleaved into CTFβ in an intracellular environment under the action of β-secretase, one of which is known as β-site APP cleaving enzyme-1 (BACE-1). BACE-1 inhibitors may help to treat AD by blocking this process. LY2811376 (3), an oral BACE-1 inhibitor showing superiorities in pharmacokinetics and pharmacology in the rat model, was terminated in phase 1 because of its retinal toxicity. Its followed drug named LY2886721 (4) failed due to hepatotoxicity in phase 2 experiments [50]. Verubecestat (5) was the first BACE-1 inhibitor to enter phase 3 clinical trials for the treatment of amnestic mild cognitive impairment and prodromal AD, which means that it would influence the deposition of Aβ before the appearance of dementia symptoms. However, it was failed due to the invalid effect, while the cognition and daily function were even worse in the verubecestat administered group than that of the placebo to some degree [51]. Atabecestat (6) showed robust Aβ reduction and safety in preclinical models [52] but failed for severely elevated liver enzymes [53]. Lanabecestat (7), an oral BACE-1 inhibitor, showed good clinical safety but the phase 3 trials were terminated due to the lack of ability to improve cognition. Some natural products with the representative examples of berberine, baicalein, and myricetin also showed potential in this respect, as they affect BACE-1 in multiple ways by not only inhibiting enzymic activity but also suppressing gene expression [54]. There is still a long way before those natural products are developed into therapeutic drugs. Although clinic trials of BACE-1 inhibitors failed on safety grounds or could not achieve the desired effect until now, but it’s still promising for its specific mechanism of blocking the cleavage of APP.

Monoamine oxidase (MAO) could also influence the cleavage of APP by adjusting the γ-secretase [55]. There have been several marketed monoamine oxidase inhibitors (MAOI) such as Rasagiline (8) and Ladostigil (9) used for the treatment of Parkinson's disease (PD), AD, and depression, which showed a degree of neuroprotective effects [56, 57]. Amongst them, ladostigil was designed from the pharmacophores of rasagiline and rivastigmine (10), an inhibitor of both acetyl-cholinesterase (Ache) and butyrylcholinesterase (Bche) and showed multi-target action. Based on the research of MAOI, a structure-activity relationship was found that an N-terminal substituent of propargyl can empower the structure to irreversible inhibit the MAO [50].

As for the branch of δ-secretase, there is more limited drug research than that of β- and γ-secretase. An orally bioactive δ-secretase inhibitor (11) was identified by the high throughput screening. It showed good permeation of the blood-brain barrier in mice models and negligible toxicities through long-term administration [58, 59], while its clinical effect remained to be studied. It’s also reported that δ-secretase improves the level of Aβ by the degradation of TrkB. 7,8-dihydrxoflavone (12) and its prodrug (13) are the agonists of TrkB, which have shown success in the treatment of AD [60, 61]. The δ-secretase inhibitor is a potential multi-target therapy aimed at blocking the cleavage of APP and TrkB, which might break the ice of the AD therapy situation.

As an age-related disease, metabolic syndrome (MetS) was proved to be linked to increasing the risk of AD. Many shreds of evidence showed that Aβ deposition was associated with obesity, diabetes, cholesterol levels, and hypertension. It was observed that Aβ accumulation was accelerated in elevated fasting glucose and blood pressure patient [62]. Metformin, a drug widely used for the treatment of type 2 diabetes mellitus, could reduce the level of BACE-1 in mice models which indicated that it worked by inhibiting the cleavage of APP [63, 64]. A phase 2/3 clinical trial has been conducted to preventAD by metformin.

In non-amyloidogenic pathway, APP is digested by α-secretase into sAPPα with the fracture of the Aβ sequence, which could competitively consume the content of APP and inhibit the β-secretase enzymolysis [32]. APH-1105 is a newly-developed α-secretase modulator with few reports, which has been currently entered phase 2 clinical trials [3]. ID1201 is extracted from the fruit of *Melia toosendan* Sieb. et Zucc, which can enhance the non-amyloidogenic metabolism by activating α-secretase [65]. In one investigation conducted on 5Xfamilial AD (FAD) mice, ID1201 treatment reduced insoluble Aβ_42_ and increased sAPPα level, indicating that it could be used to treat AD [66]. In addition, rivastigmine (10), a cholinesterase inhibitor, can also guide the cleavage process of APP away from β-secretase and towards α-secretase, apart from its cholinesterase inhibition [19].

### 3. Anti-aggregation of Aβ

Amyloid-binding alcohol dehydrogenase (ABAD), which mainly exists in mitochondria, acts as a transformer of the estrone to estradiol progress [67, 68]. ABAD will bind to Aβ specifically at a micromolar concentration which promotes the aggregation of Aβ and thereby leads to neurotoxicity. Based on this, ABAD-Aβ interaction inhibitors were developed for the treatment of AD through anti-aggregation of Aβ. It was reported that frentizole (14), an FDA-approved immunosuppressive drug, was found to be a potent inhibitor of the Aβ-ABAD interaction. Based on the structure of frentizole, a series of benzothiazole urea derivatives were synthesized and evaluated. Amongst them, two compounds (15, 16) showed the best inhibitory activity against Aβ-ABAD interaction and the potential to penetrate the blood-brain barrier [69]. However, the IC_50_ values of these compounds were unable to be determined due to the limited solubility.

Metal chelators are a special type of agent hindering the aggregation of Aβ. In a physiological environment, metal ions such as Fe^3+^, Cu^2+^, and Zn^2+^ could bind to Aβ to facilitate its aggregation [70-72]. Metal chelators could chelate the metal ions and obstruct the Aβ aggregation progress. Clioquinol (17), an anti-infective drug, was found to inhibit Aβ aggregation induced by Cu^2+^ and Zn^2+^, but the evidence of cognitive improvement was deficient and the clinical trials failed due to toxicity [70]. Deferiprone (18), a potent iron chelator, has entered phase 2 clinical trials for prodromal and mild Alzheimer's disease [12, 72]. As outlined above, metal chelators reduce Aβ aggregation by capturing metal ions, which will also affect the normal dynamic balance of metal ions and cause inevitable side effects. Adjustment of the affinity and selectivity, as well as combination with drugs of different mechanisms, were the improvement strategies of metal chelators.

Some natural products also showed activity of Aβ aggregate inhibition. Curcumin (19) and rosmarinic acid (20) were reported for their inhibiting ability of Aβ aggregation *in vitro*, and it was conjectured to be associated with their compact and symmetric structure. The anti-AD ability of the extractive of *Crocus sativus* has been proved in several randomized and double-blind clinical trials with similar effects and lower toxicity compared with donepezil and memantine. Research showed crocin was the main effective constituent of *Crocus sativus*, which could inhibit the aggregation of Aβ and downstream neurotoxicity. Natural products provide novel molecular skeletons and always possess multiple mechanisms [73].

### 4. Promoting Aβ clearance

Some strategies aimed at Aβ clearance, include promoting the metabolism of Aβ or removing Aβ by the antibody. The tyrosine kinase Abelson (Abl) participates in a wide range of daily physiological activities. The degree of tyrosine phosphorylation can be increased by Abl in the hippocampus and entorhinal cortex of Alzheimer's patients. It was reported that inhibition of Abl prevented Aβ_1-42_ fibrils caused cell death. On the other hand, the levels of Abl were increased by the hippocampal injection of Aβ fibrils, which indicated that Abl might participate in regulating the clearance of Aβ. Based on this hypothesis, some drugs attempted to inhibit Abl to facilitate the clearance of Aβ. Nilotinib (21) is one antineoplastic currently used clinically for the treatment of chronic myelogenous leukemia, which also functions as an Abl inhibitor. One study has determined the mechanisms of parkin-beclin-1 interaction induced by nilotinib and demonstrated that the Aβ clearance was promoted. Specifically, parkin is an E3 ubiquitin ligase that interacts with beclin-1 and enhances amyloid clearance. Inefficient ubiquitination of AD patients will cause the decreased parkin-beclin-1 interaction, while nilotinib could improve the levels of endogenous parkin by inhibiting the tyrosine-phosphorylation of parkin, thereby promoting the self-ubiquitination and Aβ clearance [74, 75].

Immunotherapy seems to be the most promising strategy aiming at Aβ clearance so far. It was first reported that the level of Aβ was decreased by administrated immunotherapy in 2002, but the cognitive improvement didn't appear with it [76]. Even worse, there were 6% of patients suffered from meningoencephalitis [77]. Later, a powerful class of passive immunotherapy, monoclonal antibody (mAb) come out and dominated. Different mAbs were developed aimed at different phases of Aβ, such as crenezumab, donanemab, and gantenerumab recognizing oligomers, solanezumab directed at monomers, lecanemab aimed at soluble protofibrils and aducanumab mainly capturing plaque and oligomers [3, 72, 78, 79]. Amongst them, aducanumab was the first approved mAb for AD treatment by FDA in 2021 [16]. It claimed that aducanumab could alter the disease progression rather than only alleviate the symptom. However, evidence for this claim is still lacking based on existing clinical outcomes. Moreover, the accelerated approval pathway and surrogate endpoint were controversial [80]. A further randomized controlled clinical trial would be conducted and will be completed by 2030 [16].

### 5. Neuroprotection

After the Aβ oligomer's emergence, neurotoxicity increases dramatically. Some drug developments focus on decreasing neurotoxicity. Sigma-2 receptor complex takes part in the regulation of cellular damage response, and it was demonstrated that antagonizing the sigma-2 receptor complex would contribute to blocking neurotoxicity of Aβ oligomer. CT1812 (22), a sigma-2 receptor complex antagonist, showed remarkable anti-AD activity in mice models and orally bioavailable, brain penetrant, safety, and well tolerance in healthy volunteers. CT1812 could displace Aβ oligomers binding to synaptic receptors significantly and dose-dependently to protect neurons from neurotoxicity. In other pathways, it can increase the number of synapses and expression of the protein in neurons, thereby improving cognition. According to the observations of the clinical cases, CT1812 can reverse the expression of AD-related proteins dysregulation and reduce the concentrations of phosphorylated tau fragments, which is associated with another mainstream hypothesis of AD [81]. Sigma-1 receptor plays a major role in stress response mechanisms of mitochondria and endoplasmic reticulum. The mitochondrial protective function of the sigma-1 receptor has been proved *in vivo* [82]. Blarcamesine (23), a tetrahydrofuran derivative, is a sigma-1 receptor agonist and the ligand of muscarinic receptor. It showed neuroprotective, anti-mitochondrial damage and anti-amnesic activities in mice models. It has completed phase 2 clinical trials in 2020 and begun phase 3 clinical trials [12, 83]. Moreover, some other sigma receptors modulators are currently underway in phase 2 clinical trials for AD treatments such as Edonerpic (24).

Low-dose levetiracetam (25), an anti-epileptic drug, was found to reduce Aβ-induced neuronal hyperactivity and improve synaptic function. The clinical trial of phase 3 for this drug was expected to end in 2022 [12]. Sumifilam (PTI-125) could stabilize the interaction of soluble Aβ and the α7-nicotinic acetylcholine receptor (α7nAChR) to prevent toxic signaling of Aβ, which improved multiple biomarkers of AD in phase 2 studies [84]. Some endogenous neurotrophins also showed protecting activity against Aβ-induced neurotoxicity and they might possess more security. For instance, brain-derived neurotrophic factor (BDNF), a kind of decreasing neurotrophins in AD patient brains [85-87], can prevent Aβ-induced neurotoxicity and improve learning and memory abilities in AD animal models [88]. Besides, BDNF can also inhibit the generation of Aβ [89]. One strategy for promoting BDNF gene delivery was regarded as a potential treatment [24]. These types of therapies are aimed at protecting the health of brain neurons rather than obstructing the amyloidogenic pathway, which may be a common strategy for the treatment of AD induced by different factors.

## 6.Drug development issues and challenges

### 1. Inequivalence between anti-amyloidogenic pathway and anti-AD

The attempts of AD medication that target the amyloidogenic pathway had a low success rate so far. One of the common problems is the inequivalence between the direct effects of the amyloidogenic pathway and the improvement of clinical symptoms. Some therapies in clinical trials have exhibited certain effects on decreasing the level of Aβ oligomer or relative biomarkers but failed in reaching clinical endpoint [90]. USA National Institutes of Health (NIH) Biomarkers Definitions Working Group defined a term called surrogate endpoint to substitute for a clinical endpoint [91], which helped to shorten the time as well as decrease the cost and risk of clinical trials. However, it seems tough for biomarkers to define a surrogate endpoint in the amyloidogenic pathway based on therapeutic and pathophysiologic research, hence left alone clinical endpoint. More research and clinical trials are needed to support whether the strategy of the anti-amyloidogenic pathway significantly and steadily improves the symptoms of AD.

### 2. Differences between animal models and clinical trials

Some candidate drugs showed prominent activities in animal experiments but fell short of target in clinical trials. This incongruity might occur due to the discrepancy between humans and laboratory animals in genetic background. It was reported that the paroxysm of AD in most animal models is on account of gene mutations involved in early-onset familial AD, which account for only 1% in cases of humans [20]. More importantly, there are differences in physiological and pathogenic structure between humans and animals [92-94]. The human cerebral cortex, compared with mice, possesses conservative basic structure and cortical development while the area and number of neurons increased 1,000-fold [95-97]. Besides, some specialized features were confirmed in the human brain, such as the presence of interlaminar astrocytes and rosehip neurons and the expansion of superficial cortical layers [98-100]. The difference in gene transcriptional regulation in neuronal structure and function areas also cannot be ignored [92]. Therefore, it’s necessary to clarify the pathogenesis of AD to develop better models based on the common features of animals and humans.

### 3. Limitations of single target administration

The targets for AD treatment are diverse and appealing [101], but few of these therapies aimed at a single target reach the anticipated curative effect. The single-target only meets the unilateral goal. As the amyloidogenic pathway is reticular and complex, it might be insufficient for the inhibition of a single step to interdict the whole amyloidogenic pathway. Meanwhile, the single block of the amyloidogenic pathway seems inadequate as the pathogenesis of AD is extremely complicated. Multitarget therapy gradually shows advantages, especially fitting for AD as it’s a multifactorial neurodegenerative disease. Multi-target-directed ligands (MTDLs), which were designed by a combination of structurally active pharmacophores, were attractive strategies for AD therapy. For example, a series of tacrine derivatives, which are designed from the cholinesterase inhibitor assembled with the property of Aβ aggregation, acted as active dual inhibitors of both and possess the potential to be an MTDLs for AD treatment. The drugs act on multiple targets of the amyloidogenic pathway and numerous other pathways simultaneously might be the way out of the AD therapeutic dilemma.

### 4. Side effects caused by complex downstream effects

Some clinic trials of AD therapy were halted on account of the intolerable toxicity, partly because of off-target effects. More commonly, even though a candidate drug interacted with a certain target in high specificity, the downstream effects of that target are complex, which might lead to unexpected side effects. For example, inhibition of γ-secretase not only counters Aβ production but also influences the Notch signaling pathway [102], which resulted in severe side effects. In addition to γ-secretase, β- and δ-secretase also have various substrates and biological functions. In fact, some clinical trials of secretase inhibitors have been suspended because of severe toxicity and side effects [12]. To reduce the degree of side effects, the mechanism difference between the amyloidogenic pathway and normal physiological pathway as well as the structure-activity relationship require clarification.

### 5. The continuity and irreversibility of nerve damage

It’s reported that the absence of curative effect in clinical trials might be due to insufficient reduction of Aβ level for the heavy neurodegeneration [103]. The brain nerve of AD patients had suffered from irreversible damage once the event of Aβ accumulation happened. By the time a patient developed symptoms of memory and cognitive decline, nerve damage had progressed to a certain degree. Decreasing the level of Aβ may only protect the nerves from new damage, but can’t reverse the injuries that have already occurred. And the downstream influences such as inflammatory factors and apoptosis will still last for a while even if Aβ has been eliminated. A combination of early intervention and medication could be promising strategies for the prevention of neurotoxicity.

### 6. Incomplete cognizance of amyloid cascade hypothesis

The pathogenic mechanism of Aβ is still not completely clear since Aβ was discovered at the start of the last century [20], which become one of the biggest difficulties for the research of Aβ-targeted drugs. Yet most of the evidence so far suggests the amyloid cascade hypothesis still makes sense, especially in the condition of a pre-symptomatic phase of AD or in the very mild cognitive impairment stage [53]. During the early phase of Aβ deposition, the effects of amyloid modification remind to be trialed [104]. It’s estimated that the accumulation of Aβ had sustained in the brain for 10 to 15 years before AD symptoms [103]. The long incubation periods of nearly 30 years exist in some cases with multiple pathological changes in the amyloidogenic pathway [105]. Based on the frameworks of the National Institute on Aging (NIA) and the Alzheimer's Association, preclinical phases and prodromal AD were regarded as two stages before mild dementia, which meant brain changes without symptoms and mild cognitive impairment (MCI) [106]. Research aiming at early intervention and prevention of AD through the amyloidogenic pathway might achieve breakthrough rather than late treatment. Furthermore, some researchers hold the view of lifelong management of Aβ metabolism [103], which regarded Aβ as another form of cholesterol or blood glucose.

## 7.Conclusion and Prospect

The amyloid cascade hypothesis has come up for a long time compared to other hypotheses, but the understanding of this pathway is still shrouded in mystery. Fragmentary and outdated understanding cannot match up with this complex pathway. New neurotoxic factors, branches, and secrets have been uncovered one after the other, advancing the research on Alzheimer's disease treatment. In this review, existing discoveries and recent conjectures of the Aβ-induced pathogenesis were in-depth summarized, classified, and described. The therapeutic strategies aimed at potential targets were systematically enumerated and analyzed. Although scarcely any of these attempts met desired intentions, establishing empirical laws from failures were necessary.

With the development of research, people's cognition of the amyloid cascade hypothesis was constantly refreshed. According to reports, large amounts of Aβ were also discovered in the brains of almost one-third of cognitively normal elderly people [107, 108]. The production and clearance of Aβ are keeping a balance, while the balance is weak in a pathological state. Breaking this balance imprudently might activate the feedback mechanism and accelerate the course of the disease, which might be one reason for some drugs showing worse effects than placebos in clinical trials. Some researchers begin to suspect the guiding significance of the amyloid cascade hypothesis for AD treatment, especially when more and more attempts move towards failure. Nevertheless, more scholars still hold a positive attitude towards adjusting Aβ levels in AD patients’ brains. Encouragingly, some attempts in clinics are sanguine [90].

Nowadays, the significance and complexity of the amyloid cascade hypothesis have been undisputed. How to achieve the goals of treatment is still a challenge for medical researchers and drug developers. Immunotherapy seems to be the most potent strategy so far as aducanumab has been proved by FDA in 2021 although it’s a controversial accelerated approval [16]. In contrast, the development status of small molecule drugs aiming at the amyloid cascade hypothesis is not optimistic, but it doesn't mean hopelessness. In addition to discovering new molecules with occupancy-based pharmacological activity, is also promising to induce the degradation of important proteins by difunctional molecules such as proteolysis-targeting chimera (PROTAC) [109] and autophagy-targeting chimera (AUTAC) [110], which is known as targeted protein degradation (TPD) technology. However, the precondition for TPD is discovering specific ligands of important proteins of the amyloid cascade hypothesis. Plenty of evidence support that the amyloid cascade hypothesis is not an isolated pathway that demands constant attempts of multi-mechanism and multi-discipline.
